# Integrating Hormesis and Xenohormesis in Plants Through Fitness and Response Variables

**DOI:** 10.3390/plants15182770

**Published:** 2026-09-10

**Authors:** Marcela Vargas-Hernandez, Samantha de Jesus Rivero-Montejo, Perla Valeria Munguia-Fragozo, Diana Maria Amaya-Cruz, Erik Gustavo Tovar-Perez, Rosalia Virginia Ocampo-Velazquez, Israel Macias-Bobadilla, Irineo Torres-Pacheco

**Affiliations:** 1 Faculty of Engineering, Campus Amealco, Autonomous University of Queretaro, Carretera Amealco Temazcaltzingo, km 1, Centro, Amealco de Bonfil 76850, Queretaro, Mexico; marcela.vargas@uaq.mx (M.V.-H.); samantha.rivero@uaq.mx (S.d.J.R.-M.); valeria.munguia@uaq.mx (P.V.M.-F.); diana.amaya@uaq.mx (D.M.A.-C.); erik.tovar@uaq.mx (E.G.T.-P.); 2 Laboratory of Biosystems Engineering, Faculty of Engineering, Campus Amazcala, Autonomous University of Queretaro, Carretera a Chichimequillas, km 1 S/N, El Marques 76265, Queretaro, Mexico; rosalia.ocampo@uaq.mx

**Keywords:** stress tolerance, bioactive compounds, adaptive response

## Abstract

Hormesis is an adaptive response empirically characterized by a biphasic dose–response pattern, in which low or moderate levels of stress induce stimulatory responses whereas higher levels result in inhibitory or toxic effects. Although the biphasic dose–response pattern provides an empirical basis for identifying hormesis, it does not necessarily establish the adaptive significance of the observed response. Individual physiological, biochemical, or molecular variables may exhibit transient or ontogenetically dependent responses; therefore, fitness-related outcomes may provide an integrative criterion for evaluating the biological consequences and adaptive significance of hormesis. Relatively few studies have simultaneously evaluated, under the same stress factor applied across a defined dose or intensity gradient, both the hormetic performance of plants and the xenohormetic potential of bioactive compounds induced by this stress. Furthermore, this approach is insufficient to explain the complexity of the hormetic phenomenon. Therefore, there is a need to more precisely define how hormesis should be evaluated. This may be achieved by incorporating fitness-related variables, such as survival, growth, and reproductive capacity, together with response variables associated with the physiological, biochemical, and molecular mechanisms involved in stress adaptation, thus providing a framework which may be conceptually extended to xenohormesis. Direct evidence linking stress-induced metabolites to improved consumer fitness remains limited, precluding their interpretation in terms of xenohormetic potential.

## 1. Introduction

Throughout their evolution, the sessile nature of plants has driven the development of sophisticated defense mechanisms against various types of biotic and abiotic stress, both natural and anthropogenic. To survive and thrive under rapidly changing environmental conditions, plants have refined processes such as hormesis, which allow them to detect low levels of stressors and activate adaptive responses that enhance their resilience [1]. Although several definitions of hormesis have been proposed (Table 1), following recent conceptual developments, hormesis can be described as a nonlinear adaptive process by which cells—and, consequently, living organisms—respond to increasing stressor doses and exposure times. Under low or moderate doses and/or short exposure times, the stimulus activates signaling and transduction networks that modulate gene expression, redox metabolism, and the synthesis of protective molecules, promoting beneficial biochemical and physiological adjustments up to a certain maximal level of adaptation [2]. Once a critical threshold of dose and/or exposure time is exceeded, these defense mechanisms become saturated or dysregulated and the beneficial response diminishes, leading to toxic or inhibitory effects. This process represents a form of adaptive response that may contribute to evolutionary adaptation as it can alter the dynamic equilibrium of an organism’s normal ontogenetic state, triggering a beneficial adaptive response that results in an altered phenotype [2].

The concept of hormesis has evolved from an initial emphasis on dose toward an interpretation increasingly focused on adaptation, thereby bridging molecular-level insights with the broader concept of hormesis (Table 1). Early definitions highlighted the importance of dose in determining whether exposure resulted in beneficial, inhibitory, or toxic effects. Subsequently, hormesis was described as a stimulatory effect induced by subinhibitory doses of potentially toxic agents. More recently, emphasis has shifted toward the biphasic dose–response relationship, encompassing U-shaped or inverted U-shaped responses. Adaptation and compensation have also been incorporated as key features, consolidating hormesis as an adaptive biological process. Building on this conceptual evolution, this review proposes a framework linking stress exposure and regulatory mechanisms with physiological, biochemical, and molecular responses, ultimately connecting these responses to outcomes related to biological fitness.

Hormesis is considered a form of compensation in which exposure to low levels of stress induces beneficial functional, metabolic, or phenological effects that exceed the previous baseline state, typically by 30–60% above control values, as frequently reported for hormetic responses [12]. Hormesis, priming, and transgenerational stress memory are distinct but potentially interconnected stress response phenomena. A low or moderate stress exposure may induce a hormetic response and may also act as a priming stimulus; however, priming requires evidence of an altered response to a subsequent stress exposure. Similarly, hormetic exposure may contribute to stress memory; however, transgenerational stress memory requires evidence that stress-induced responses persist beyond the generations directly exposed to the initial stress [13]. Hormesis has been extensively studied in toxicology; however, in plants, the mechanisms underlying hormetic stimulation remain poorly understood [14]. Some authors have suggested that hormetic stimulation of plant growth is associated with increased photosynthetic efficiency [15].

Additionally, xenohormesis has been proposed as an extension of hormesis across biological levels, including plants, animals, fungi, bacteria, and protozoa [16], and as an interorganismal signaling hypothesis. Regarding xenohormesis, it has been proposed that a consumer may detect bioactive metabolites generated through the hormetic response of another organism and adjust its physiology accordingly; for example, in the plant context, environmentally stressed plants produce bioactive compounds that may influence stress resistance and survival in animals that consume them [17]. During coevolution, plants have developed secondary metabolites to protect their integrity against herbivores. In turn, herbivores have evolved behavioral strategies and biological mechanisms—such as selective feeding and detoxification enzymes, including cytochrome P450s (CYP450)—that allow them to neutralize plant phytochemicals [18]. The specialization of plant–animal interactions illustrates the potential for plant defensive compounds to contribute to adaptive responses in consumers [19]. Stress-induced plant secondary metabolites exhibit multiple bioactivities (e.g., antioxidant, anti-inflammatory, antimicrobial, and neuroprotective effects) and may influence human health, stress resistance, and survival. However, these biological activities alone do not provide definite evidence that these compounds function as informative xenohormetic signals. Because plants cannot escape environmental stress, they have evolved defensive mechanisms such as increasing the production of protective compounds that enhance their competitiveness. Therefore, under stress conditions, plants may increase the production and accumulation of bioactive compounds as part of their defense responses and, as such, controlled stress may provide an opportunity to enhance specific bioactive compounds with potential nutraceutical value [17].

The aim of this manuscript is to review current approaches and provide an up-to-date perspective on hormesis and xenohormesis, with particular emphasis on variables related to biological fitness. We present research findings in which plants are exposed to a single stressor across a defined gradient of dose or intensity, with particular emphasis on studies that simultaneously evaluate plant hormetic responses and the xenohormetic potential of stress-induced bioactive compounds within the same biological system. This approach provides insight into how adaptive responses in plants are associated with the production of metabolites that may confer biological benefits to consuming organisms. Furthermore, reviewing these studies enables the identification of key variables currently used to describe and characterize hormetic and xenohormetic responses in plant models.

The main novelty of this review lies in the proposal of a conceptual framework that connects stress-activated regulatory responses; physiological, biochemical, and molecular response variables; and their ultimate consequences for biological fitness throughout the hormetic process. For operational purposes, the framework distinguishes the following three levels of evidence: biphasic dose–response patterns provide empirical evidence for the identification of hormesis; physiological, biochemical, and molecular measurements enable the exploration of its underlying mechanisms; and survival and reproductive success allow for assessment of the associated consequences for biological fitness. Unlike approaches that characterize hormesis primarily on the basis of biphasic dose–response patterns in individual variables, such as growth, we propose distinguishing the empirical identification of a hormetic response from the evaluation of its underlying mechanisms and adaptive significance. From this perspective, hormesis is understood as an adaptive process emerging from the coordinated interaction among regulatory responses, physiological adjustments, and their consequences for biological fitness. Therefore, fitness-related outcomes—including survival, reproductive capacity, and offspring quality—are relevant for elucidating the roles of hormesis in the adaptive process, even though their assessment may pose significant experimental challenges.

A second novel contribution of this review is the explicit integration of hormesis and plant xenohormesis within a unified conceptual framework. In this context, this review examines how stress-induced changes in plant bioactive compounds may connect the adaptive responses of stressed plants with subsequent biological responses in consuming organisms. This perspective enables the identification of a potential functional continuity between stress-induced metabolic changes in plants and their biological effects across trophic levels. Thus, rather than defining hormesis solely based on biphasic dose–response patterns or growth stimulation, this review adopts a perspective centered on biological fitness, explicitly considering the trade-offs among growth, defense, stress tolerance, and functional performance in relation to organismal adaptation and fitness.

## 2. Literature Selection Strategy

A structured literature search was conducted following relevant PRISMA 2020 [20] reporting recommendations to identify studies evaluating hormetic responses in agriculturally important crops together with the induction or accumulation of secondary metabolites or other bioactive compounds under the same stressor. Searches were performed in Web of Science, Scopus, PubMed, ScienceDirect, and Google Scholar, covering all publications available up to March 2026, which was the date of the final literature search. The search strategy included combinations of the terms “hormesis”, “plant hormesis”, “crop”, “agricultural crop”, “hormetic response”, “dose-response”, “inverted U-shaped response”, “low-dose stimulation”, “plant stress”, “elicitor”, “secondary metabolites”, “bioactive compounds”, “chlorophylls”, “carotenoids”, “phenolic compounds”, “flavonoids”, “flavonols”, “anthocyanins”, “glucosinolates”, “terpenoids”, and “xenohormesis”, using the Boolean operators AND and OR. Search strings were adapted to the syntax of each database.

## 3. How Are Hormesis and Xenohormetic Potential Evaluated and Reported in Plants?

To date, hormesis studies have predominantly focused on yield-related variables (e.g., growth, biomass, and productivity), whereas mechanistic investigations of metabolic pathways and biochemical processes—particularly those involved in plant defense systems—remain limited. Furthermore, relatively few studies have evaluated a single stressor across a full dose gradient that elicits a hormetic biomass response—i.e., growth or yield stimulation at low doses followed by inhibition at higher doses—while simultaneously documenting changes in bioactive compound production. In addition, a small number of investigations have either constructed a hormetic dose–response curve for bioactive metabolites or provided molecular evidence (e.g., genes, enzymes, or pathways) directly linked to the initial stimulatory phase of hormesis along the same stress gradient. Most xenohormesis-related reviews have emphasized the human health benefits of secondary metabolites. As detailed in Table 2, plant hormesis is directly linked to the increased production of xenohormetic secondary metabolites under low-intensity stress; however, this aspect remains underexplored. Hormetic reports mainly utilize elicitor-type stressors (e.g., heavy metals, UV radiation, nanoparticles, salinity), and plant hormesis studies most often assess growth, redox balance, and photosynthetic activity as integrative indicators of performance [21]; for example, in *Lonicera japonica*, low cadmium doses (25 mg/L) induced hormetic responses in photosynthetic pigments (chlorophyll a, chlorophyll b, total chlorophyll, and carotenoids), while higher doses promoted pigment degradation, which may inhibit photosynthesis [22]. Photosystem II (PSII) is a pigment–protein complex in chloroplasts that drives light-induced charge separation and water splitting during photosynthesis. A separate study considering the same species under cadmium exposure conditions reported that, in addition to increasing photosynthetic pigments, Cd also enhanced stomatal conductance and increased the light-saturated net photosynthetic rate. Gas exchange responses were also observed, as indicated by higher stomatal conductance and transpiration rates. In addition, Cd increased ΦPSII and qP parameters, indicating that the majority of photons absorbed by PSII were efficiently used in photochemical processes [23]. Although PSII is essential for photosynthetic responses, it is also a significant source of reactive oxygen species (ROS), particularly when light energy exceeds its capacity for utilization. At low levels, ROS act as beneficial signaling molecules, whereas excessive ROS are harmful. In another study, antioxidant responses have been reported, including increased antioxidant activity and higher levels of SOD, POD, CAT, and APX [24].

Plant growth regulators coordinate plant growth and development. Hormetic doses of Pb and Cd increase indole-3-acetic acid (IAA) levels and reduce oxidative stress, contributing to the hormetic stimulation of shoot growth, which is also associated with increased flavonoid content [21]. SA and abscisic acid (ABA) respond to biotic and abiotic stresses and play key roles in hormetic defense responses. In edible pea (*Pisum sativum*), low concentrations of Pb induced hormesis associated with flavonoid accumulation, while high doses caused toxicity during infestation by *Acyrthosiphon pisum* [27]. The doses that induced the best response of Pb upregulated genes responsible for phytohormone biosynthesis, including 12-oxophytodienoate reductase 1 (*OPR1*) and jasmonic acid–amido synthetase (*JAR1*), as well as the ethylene biosynthesis gene 1-aminocyclopropane-1-carboxylate synthase 3 (*ACS3*), increasing jasmonates (JA/MeJA) and the ethylene precursor 1-aminocyclopropane-1-carboxylic acid (ACC). Jasmonates then act as key signals that coordinate plant development and defense against biotic and abiotic stresses [29].

As noted, relatively few studies have reported hormetic processes associated with the production of plant defense metabolites. Duarte and Sierra [33] investigated the effect of UV-C light on post-harvest broccoli (*Brassica oleracea*). They found that a dose of 1.2 kJ/m^2^ induced a hormetic response by delaying yellowing and reducing weight loss during storage. This response was accompanied by increased expression of genes such as phenylalanine N-hydroxylase (*CYP79A2*), tryptophan N-hydroxylase (*CYP79B3*), dihomo-methionine N-hydroxylase (*CYP79F1*), and flavonoid monooxygenase (*F3H1*), as well as chalcone synthase (*CHS*) and 4-coumarate-CoA ligase (*4CL*), which are involved in the biosynthetic pathways of phenylpropanoids, indole alkaloids, glucosinolates, and flavonoids. Xu et al. [32] reported that low and moderate supplemental UV-C (9.6 and 15 kJ/m^2^) in strawberries (*Fragaria × ananassa*) promoted the expression of *FaCHS1*, *FaCHI*, *FaFHT*, *FaDFR*, *FaFLS*, and *FaFGT*—key genes regulating multiple steps in flavonoid and anthocyanin biosynthesis. A moderate dose of UV-C (3 kJ/m^2^) in grapes (*Vitis vinifera* L.) induced hormetic stimulation of secondary metabolism, increasing bioactive compounds and enhancing fruit color. Table 3 classifies the compounds listed in Table 2 that accumulate under hormesis-inducing stress, summarizing their biological functions in plants and their nutraceutical/xenohormetic relevance in humans. Terpenoids, flavonoids, phenolics, and glucosinolates contribute to plant defense, redox regulation, structural reinforcement, and stress signaling, while also exhibiting antioxidant, anti-inflammatory, anticancer, cardioprotective, and neuroprotective activities. Furthermore, they promote plant survival by modulating growth and development, hormonal and physiological responses, pollinator attraction, homeostasis, and reproduction.

## 4. Associated Response Variables for Evaluating Hormesis and Xenohormesis Processes

**Figure 1:**
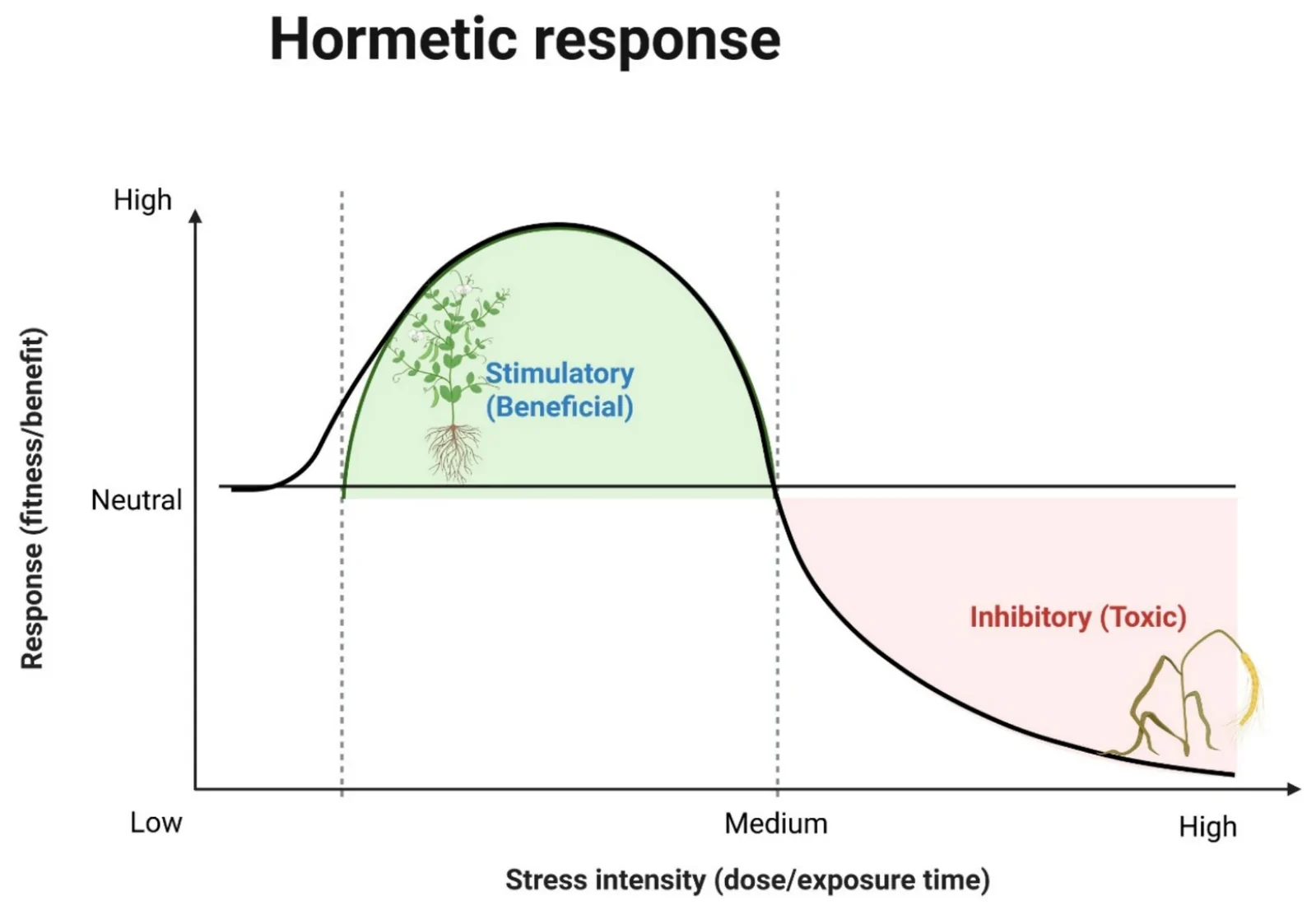
Conceptual framework of hermetic responses. The vertical dashed lines delimit the stress-intensity zones associated with the response, visually separating the stimulatory (beneficial) region from the transition toward the inhibitory (toxic) response.

It is necessary to establish appropriate criteria for measuring hormesis. While plant growth has often been used as the primary variable of the hormetic response [55], relying exclusively on growth can be conceptually restrictive. Hormesis should not be identified solely by growth stimulation; rather, it should be considered an adaptive reorganization process in which different variables may exhibit compensatory responses. We argue that hormesis should be understood frequently as an adaptive trade-off, in which increased defense or stress tolerance can be observed without simultaneous increases in growth or productivity. From this perspective, growth is not the phenomenon itself, but rather one possible response variable among others, reflecting the underlying adaptive process. These trade-offs arise from resource constraints that prevent plants from simultaneously maximizing all physiological functions, thus forcing plants to prioritize specific processes according to the environmental conditions. In this context, the concept of hormetic compensation, proposed by Erofeeva [56], becomes particularly relevant. Hormetic compensation describes a differential response in which the stimulation of certain variables or functions occurs at the expense of the suppression or stabilization of others considered less essential under stress conditions. It is important to note that hormetic responses do not necessarily translate into synchronous improvements in all plant variables. Instead, hormetic stimulation of specific traits is often accompanied by reduction or maintenance of other traits, reflecting a coordinated, compensatory adjustment at the organismal level (Figure 1). As pointed out by Erofeeva [56], hormetic trade-offs are common because low-dose stress does not uniformly improve all plant parameters. While some variables may show signs of stimulation, others may remain unchanged or even decline relative to the control. Consequently, a biphasic response in a single variable demonstrates a non-monotonic response for this specific endpoint, but its interpretation as hormesis depends on whether this variable is functionally related to fitness. Variables that contribute to survival, reproductive success, or other biologically justified proxies of fitness may provide evidence of hormesis, whereas transient molecular, biochemical, or metabolic responses without a demonstrated relationship to fitness should be interpreted as components of the stress response, rather than as specific evidence of hormesis. This interpretation also depends on the temporal and developmental context in which the variable is measured.

**Figure 2:**
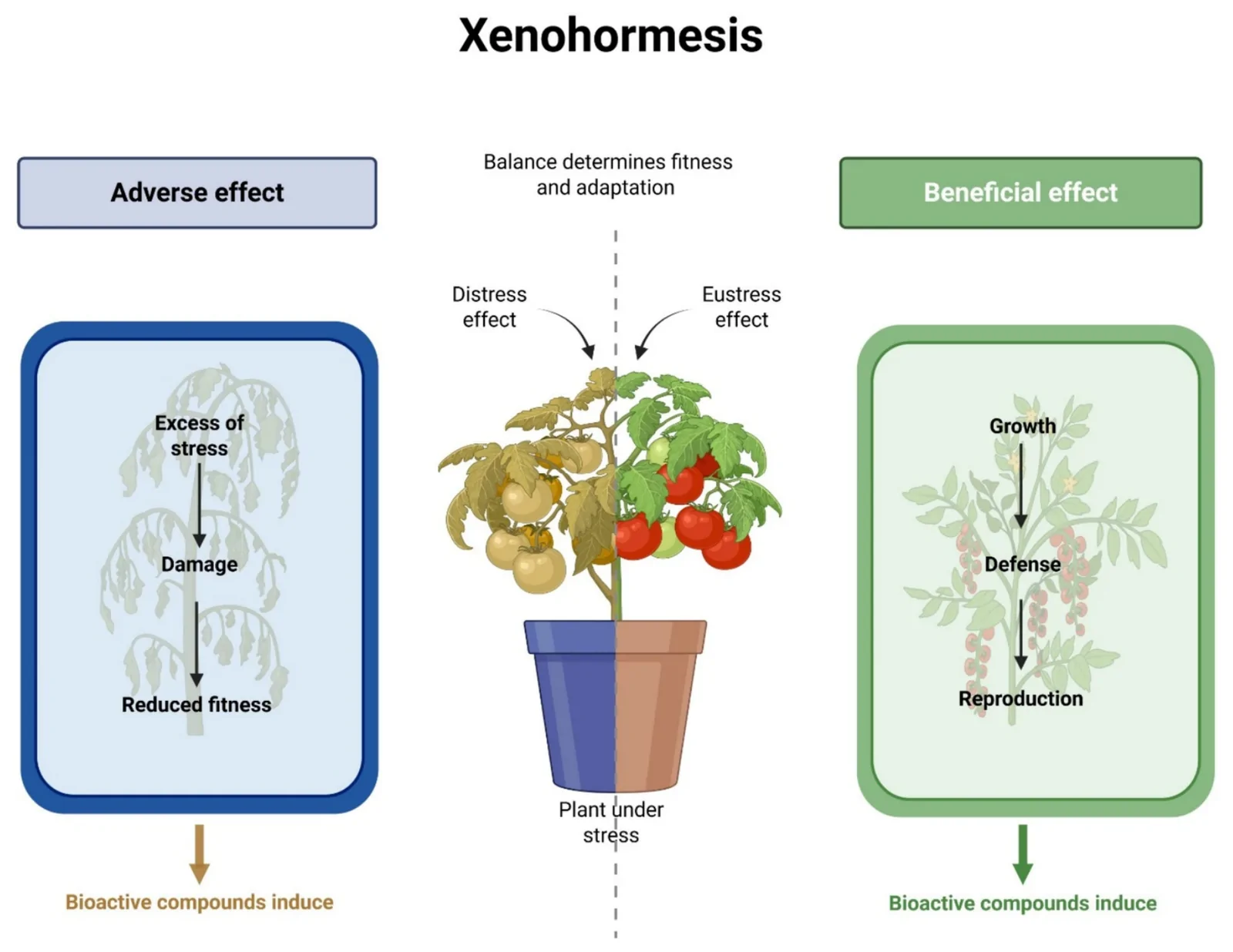
Conceptual framework of stress and outcomes in plant xenohormesis based of fitness and adaptation. Blue indicates adverse effects associated with excessive stress, damage, and reduced fitness, whereas green indicates beneficial effects associated with growth, defense, and reproduction. The arrows indicate the level at which the different responses may be progressively activated.

Growth encompasses resource allocation and regulatory adjustments that may involve trade-offs with other components of fitness, particularly defense. Growth–defense trade-offs may arise from resource constraints that influence the relative investments in growth and defense depending on internal and external conditions. For example, activation of defenses against pathogens can be accompanied by reduced growth, a pattern that may be consistent with the metabolic costs associated with defense and repair processes. However, while biomass is a practical indicator of hormetic responses, increased growth in response to a moderate stimulus does not necessarily imply improved fitness, as growth stimulation may not translate into enhanced survival or reproductive success. We consider that growth stimulation is a useful indicator of hormesis but should not be considered an exclusive or defining criterion. On the contrary, under shade or high-density conditions, elongation to reach light can occur at the expense of defensive capacity, increasing susceptibility to disease [57]. Similarly, high-nitrogen fertilization or mild hormetic stress can promote tissue expansion and elongation, but can also lead to nutrient dilution, reduced lignification, and decreased structural strength [58]. Growth–defense trade-offs have been associated with resource constraints and context-dependent investment in competing functions [59]. However, simultaneous changes in growth and defense-related variables do not, by themselves, demonstrate resource reallocation. Xenohormesis may be considered in relation to this framework, as many bioactive metabolites are synthesized and accumulate when defense mechanisms are activated. Some authors have indicated that there are situations in which activation of the defense system occurs at the expense of growth due to the high metabolic costs involved. However, increased defense-related responses should not be interpreted as evidence of improved fitness. Establishing such consequences requires appropriate measurements of resource allocation, growth costs, stress tolerance, survival, or reproductive performance (Figure 2).

When plants are exposed to a stressor, they tend to prioritize genetic, physiological, and phenological adjustments that enhance their survival. Therefore, an increase in biomass or levels of defense metabolites is not necessarily the result of hormesis. Understanding the molecular basis of these trade-offs in plants should serve as a foundation for developing breeding strategies that optimize the balance between growth and defense, thus maximizing crop yields and meeting the growing global demand for food and biofuels. Based on the above, we propose that hormesis should ideally be evaluated in terms of fitness, understood as survival, reproductive capacity (number of individuals produced), and offspring quality. In this sense, the idea that survival depends not only on growth but also on the ability to maintain defensive physiological adaptations against herbivores and pathogens is reinforced. These responses allow an organism to maintain its functionality under stress, supporting the idea that hormetic responses often prioritizes defense and resilience over biomass accumulation. In addition, response variables tend to vary depending on the stress factor that induces the response, reflecting different physiological and adaptive mechanisms according to the nature of the stimulus.

Biological fitness is understood as an organism’s capacity to survive and contribute to subsequent generations through reproductive success. Fitness is proposed as the ultimate descriptor of hormesis as, ultimately, an adaptive response should translate into an improvement in the organism’s performance or biological success. Because hormesis is conceptualized as an adaptive response, the beneficial nature of that response must be evaluated in relation to its biological and ecological context [9]. Therefore, determining whether a hormetic response represents a biological advantage requires assessing its functional relevance and its consequences for the organism’s performance. The behavior of a single physiological or biochemical variable in isolation does not demonstrate that the organism has experienced a beneficial hormetic effect. Consequently, hormesis should not be defined based on a single response variable. Plants simultaneously regulate growth, metabolism, defense, antioxidant capacity, repair, and other physiological functions, generating coordinated responses that may involve compensatory adjustments and trade-offs. Thus, one variable may increase while another decreases or remains unchanged, without implying that one response is “hormetic” and the other is not. The biological significance of hormesis emerges from the integration of these responses and their consequences for the organism’s fitness. In this sense, hormesis can be considered the integrated result of a multivariable, compensatory process, rather than a collection of independent hormetic dose–response curves.

As an integrated measure of expected reproductive contribution, fitness can be estimated as follows:

F = S × R

where S represents the probability of survival and R the reproductive success of surviving individuals [60].

This expression is considered an operational approximation of biological fitness, rather than a universal definition of fitness. Within this framework, the relationship between stress intensity and biological fitness can be represented by a dose–response curve, where the *x*-axis corresponds to increasing stress intensity and the *y*-axis represents integrated biological fitness. When exposure to low or moderate stress intensity increases biological fitness relative to the control, whereas higher levels of exposure result in a decrease, the integrated response will exhibit an inverted U-shaped hormetic pattern.

**Figure 3:**
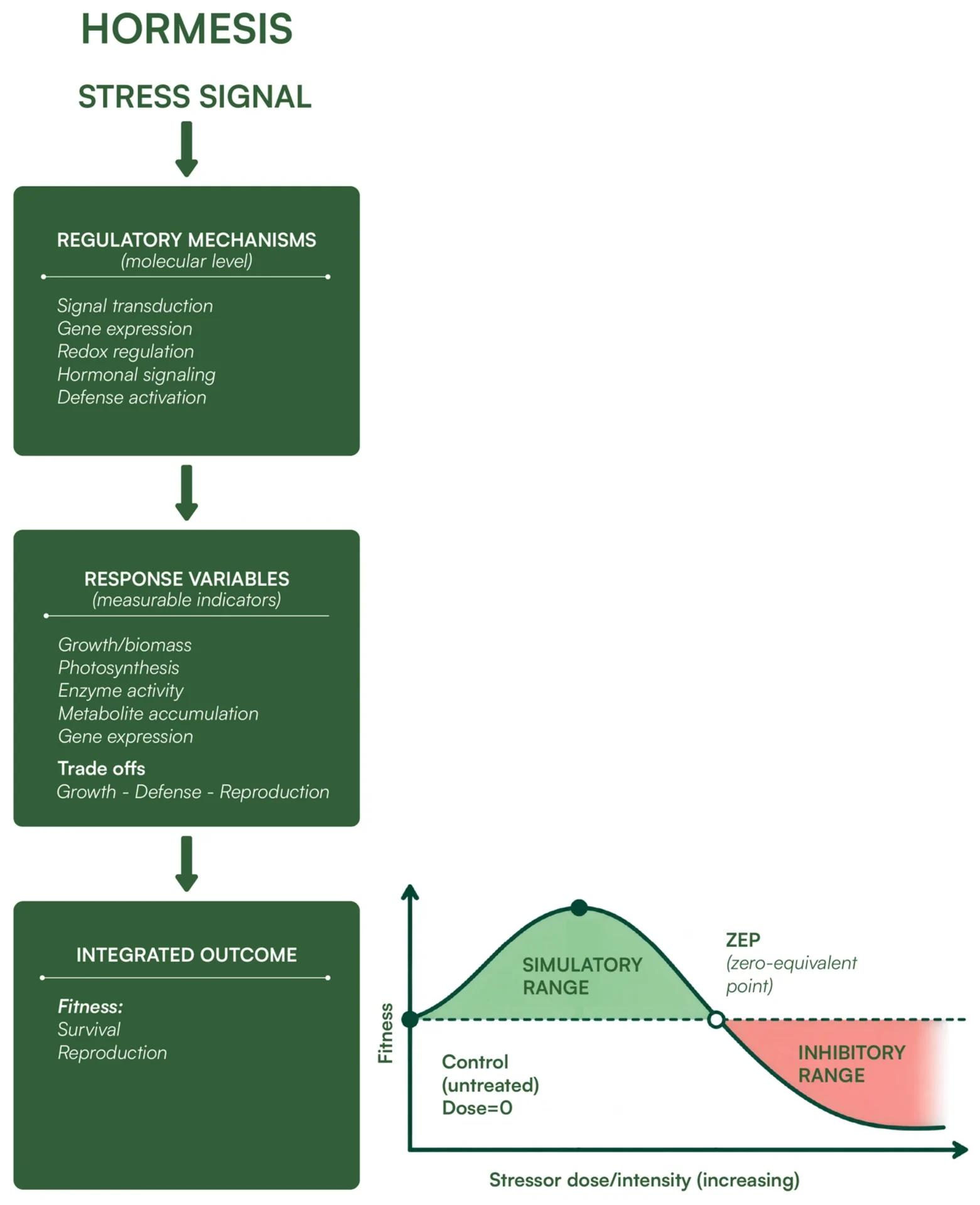
Conceptual framework integrating regulatory mechanisms, measurable response variables, trade-offs, and biological outcomes in plant hormesis.

This rationale is consistent with the conceptual framework proposed in Figure 3, in which biological fitness represents the integrated biological outcome of the regulatory and physiological processes activated by stress exposure.

One of the main conceptual challenges lies in viewing fitness as a descriptor resulting from the relationship between two fundamental components; namely, survival capacity and reproductive capacity. In this sense, survival and reproduction represent components of fitness, whereas fitness constitutes the integrated outcome of both capacities. This distinction is particularly important when incorporating fitness into the graphical representation of hormesis, since the variable to be related to the increasing stress gradient is the integrated fitness descriptor, rather than each of its components independently. Thus, the hormesis curve illustrates how stress modifies the organism’s integrated biological performance through its combined effects on survival and reproduction. On the other hand, assessing fitness experimentally poses significant practical challenges. Its evaluation often requires long-term measurements of survival, reproduction, and offspring performance, which vary substantially across plant species and life histories. Consequently, most studies rely on physiological, biochemical, molecular, or growth-related variables as practical proxies for adaptive responses. These variables should therefore be interpreted as intermediate indicators, rather than direct evidence of enhanced fitness. Thus, although fitness represents the most biologically meaningful criterion for evaluating the adaptive significance of hormesis, its experimental assessment should be tailored to the biology of each specific plant system.

Most studies implicitly interpret biphasic dose–response patterns in individual variables as evidence of hormesis, regardless of whether the measured endpoint is functionally related to fitness. However, this interpretation is conceptually limited. Response variables—such as growth, enzyme activity, metabolite accumulation, and gene expression—should not be equated with hormesis, but instead understood as measurable outputs of the regulatory processes that mediate the adaptive response. In this sense, hormesis is not defined by individual variables but by the integration of multiple regulatory mechanisms that ultimately yield a fitness-related outcome. A biphasic response in an individual variable does not, by itself, constitute evidence of hormesis unless that variable is functionally related to fitness; otherwise, it should be interpreted as a partial response of the system. Moreover, the temporal context of the response is critical. If hormesis is interpreted in relation to fitness, its strict evaluation requires endpoints measured over a biologically relevant period that allows the consequences for survival and reproductive success to be established. Therefore, transient stimulation observed at an early developmental stage should not, by itself, be considered conclusive evidence of hormesis, because its contribution to survival or reproduction may not persist throughout the life cycle. Hormesis should be interpreted as an integrative adaptive process in which multiple physiological, biochemical, and functional variables interact to determine the organism’s final state. The integrative interpretation is consistent with the view that hormesis is a coordinated response of cells and organisms to an imposed or intrinsically generated challenge that involves multiple integrative signal transduction processes, each of which is quantitatively hormetic, to coordinate a final holistic response [61]. Therefore, the strongest evidence for a hormetic response is obtained when physiological and molecular changes can be linked to improved fitness-related outcomes, such as survival and reproductive success, which represent the integrated result of multiple interacting biological processes. Although we consider fitness to be the ultimate biological outcome of a hormetic response, its experimental assessment is considerably more challenging as it requires longer observation periods and a greater number of measurements related to survival, growth, reproduction, and/or overall biological success. Consequently, most experimental studies rely on physiological, biochemical, and molecular variables as indirect indicators of hormetic responses, since these endpoints allow for detection of the adaptive adjustments induced by low-intensity stimuli in a more practical and timely manner. However, these variables do not necessarily represent hormesis itself; rather, they reflect physiological or biochemical processes that contribute to the overall adaptive response. Hormesis can be understood as a sequence of processes that begins with increasing exposure to a stress factor, which triggers the activation of various regulatory mechanisms at the cellular and physiological levels. These responses include the activation of signaling and transduction networks that modulate gene expression, redox metabolism, and the synthesis of protective molecules, allowing the organism to perceive and respond to the stimulus. As a result of these regulatory processes, measurable response variables are generated, including changes in enzymatic activity, gene expression, redox balance, metabolite concentrations, and specific physiological parameters. Some variables may provide evidence of hormesis when they are functionally linked to fitness and evaluated within an appropriate temporal context, whereas others primarily represent components of the stress response. Thus, while hormesis can be identified at the level of a specific endpoint through an appropriate biphasic dose–response, fitness provides a biologically integrative framework for evaluating the adaptive significance of hormesis at the organismal level. Survival and reproductive success integrate the net consequences of multiple measured and unmeasured responses that may act positively, negatively, or transiently throughout the life cycle (Figure 3).

It is necessary to identify the appropriate variables to assess plant fitness; for example, survival can be measured directly as the proportion of individuals remaining alive after a defined period of exposure to a specified stressor or environmental challenge. Reproductive output can be quantified as the number of viable seeds, fruits, or other reproductive structures produced per surviving individual, depending on the reproductive biology of the species. Due to the difficulty of measuring fitness, most studies measure physiological variables that reflect only part of the cellular and biochemical mechanisms underlying hormetic responses. A previous study evaluated *Hordeum vulgare* until senescence rather than exclusively during the vegetative stage. Reproductive variables, including seed number per plant, seed yield, and 1000-seed mass, were assessed together with biomass and other traits. Reproductive responses were significantly hormetic; for example, the estimated maximum response reached 118% of the control for seed number and 136% for seed yield. In addition, the F1 generation was evaluated. Offspring derived from fast-growing plants that had previously exhibited hormetic stimulation showed greater glyphosate tolerance when re-exposed. The authors interpreted these findings as evidence that low doses may enhance the fitness associated with specific stress-tolerant phenotypes [62]. In this way, hormesis can be interpreted as the emergent outcome of a chain of events linking environmental stress with internal regulatory adjustments and their physiological manifestations, ultimately resulting in an adaptive benefit for the organism. In this sense, depending on the stress factor, different physiological and molecular mechanisms underlying plant hormesis may operate; therefore, the measurable response variables can vary according to the nature of the stimulus. However, regardless of the specific stressor, we propose that fitness-related outcomes provide the most biologically meaningful framework for interpreting the adaptive significance of hormesis at the organismal level, understood in terms of survival, reproductive output, and relative fitness under defined environmental conditions (Table 4). The regulatory mechanisms presented in Table 4 should be interpreted as candidate mechanisms potentially involved in plant hormesis, rather than as established causal mechanisms. They are primarily based on physiological and molecular processes which are known to mediate plant responses to corresponding environmental stresses. However, fitness benefits associated with hormesis are often context-dependent and may only become evident under the environmental conditions in which the adaptive response provides an advantage. Thus, hormesis should not necessarily be interpreted as a universal increase in performance, but rather as an improvement in fitness-related outcomes under specific environmental constraints.

**Figure 4:**
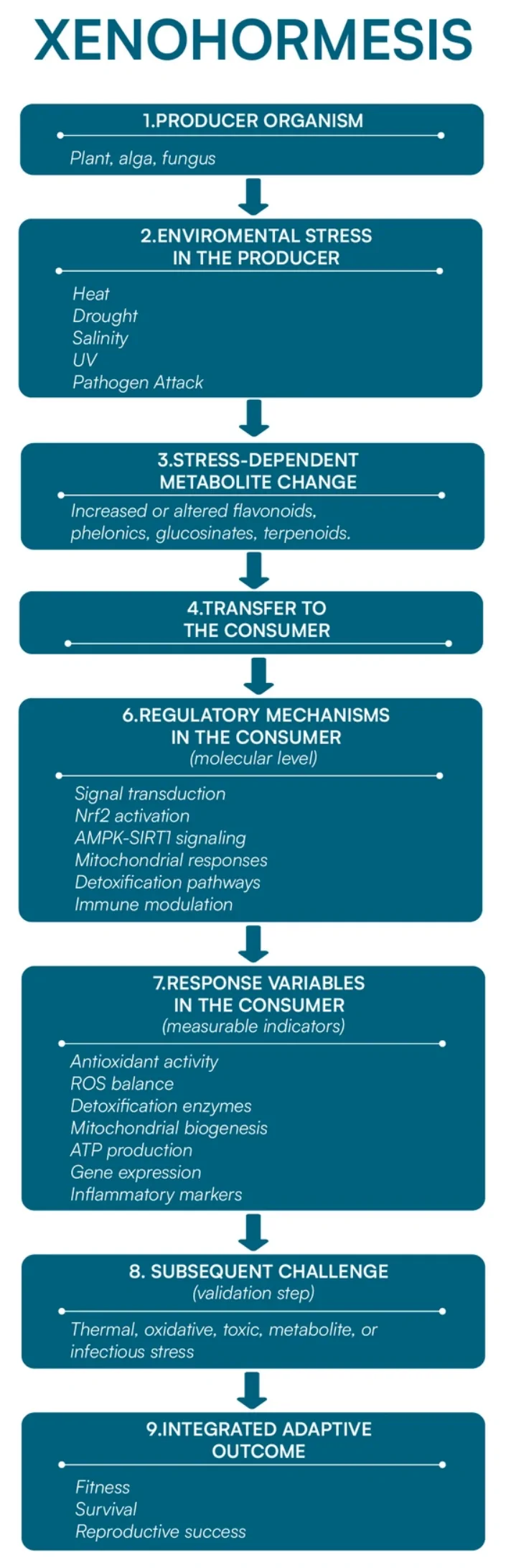
Conceptual framework describing the progression from xenohormetic signals to adaptive responses and fitness-related outcomes.

The same conceptual logic established for hormesis can also be extended to xenohormesis, although xenohormesis requires an explicit producer–consumer connection (Figure 4). In hormesis, the response results from direct exposure of an organism to a stress factor, whereas xenohormesis involves chemical signals generated by a producer organism in response to environmental stress and subsequently perceived by a consumer. A rigorous demonstration of xenohormesis therefore requires the following: (i) exposure of the producer to a defined environmental stress; (ii) a stress-dependent alteration in the concentration of a defined metabolite; (iii) transfer of that metabolite or stressed producer material to the consumer at a defined exposure level; (iv) comparison with an appropriate consumer control receiving material from non-stressed producers; (v) demonstration of an adaptive outcome in the consumer. Once transferred, these compounds may activate regulatory mechanisms related to gene expression, redox metabolism, detoxification, and other protective responses. However, such downstream effects should be considered evidence of xenohormesis only when they are experimentally linked to the stress-induced change in the producer and to a measurable adaptive benefit in the consumer, such as increased stress tolerance, survival, or reproductive performance.

It should be noted that Table 5 summarizes the candidate regulatory mechanisms and response variables associated with xenohormesis. However, these mechanisms can be broadly applied to other living organisms and at different levels of biological organization, from cellular and organ responses to individual- and population-level effects. Hormesis can occur at the level of an individual organism, although dose–response relationships are often constructed by comparing groups of independent replicates exposed to different doses. In such cases, the resulting curve represents an average response across individuals and may mask individual variability.

**Figure 5:**
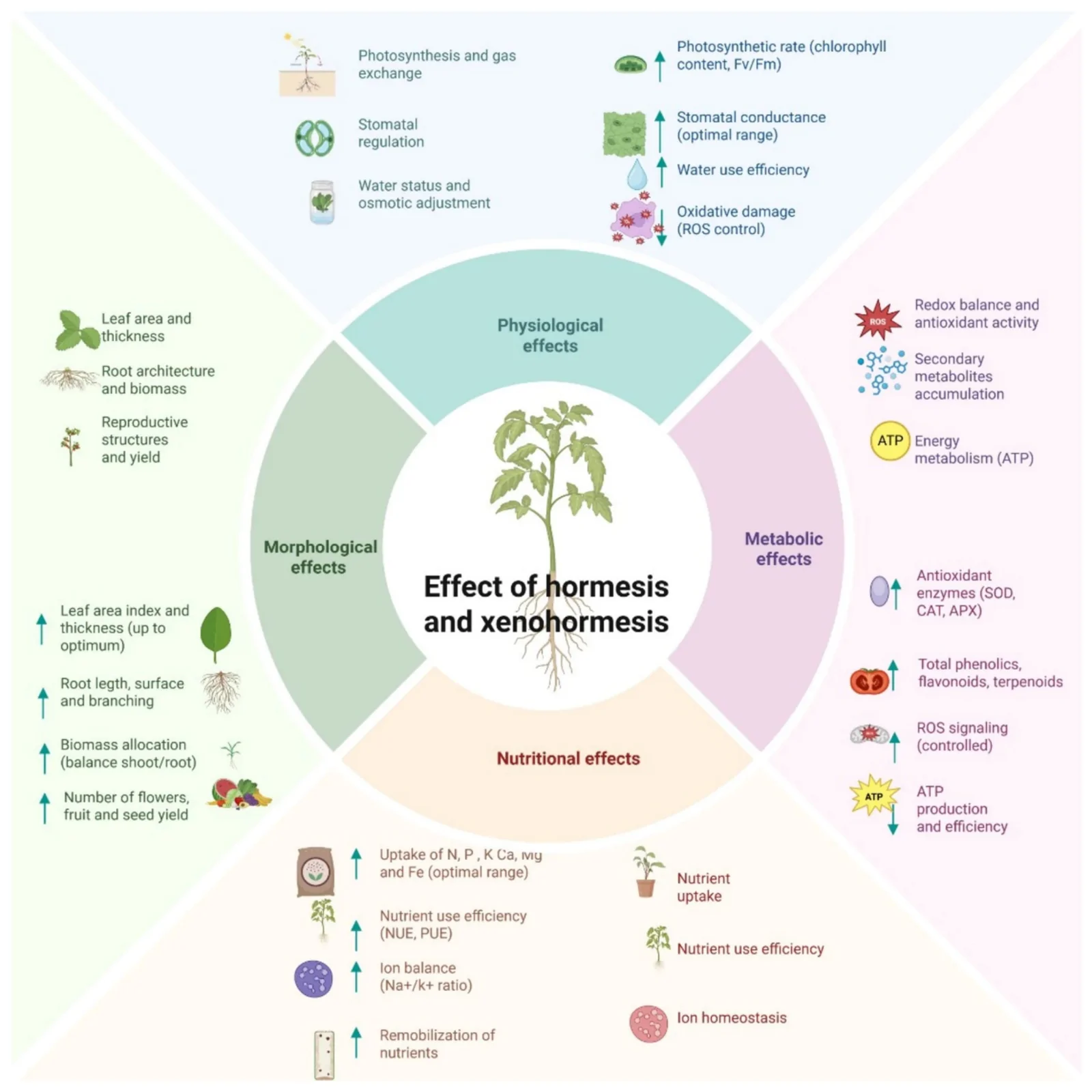
Principal effects and key components of hormesis and xenohormetic stress in relation to adaptive responses and fitness-related outcomes. The arrows indicate increases or decreases in the corresponding response variables.

When faced with a stress factor, a plant may undergo physiological, biochemical, and phenological adjustments that contribute to acclimation; however, these responses do not necessarily ensure survival or reproduction. It is also important to note that the responses presented in the tables primarily correspond to physiological, biochemical, and functional levels and are not intended to comprehensively represent genomic responses, although genomic responses are also present. Hormesis-related responses can occur over different time scales. Short-term responses primarily involve physiological and metabolic adjustments within individuals, reflecting phenotypic plasticity and acclimation to stress. In some cases, stress exposure may also induce epigenetic modifications that contribute to stress memory within the individual. Long-term responses may involve persistent epigenetic modifications, whereas the transmission of such changes to subsequent generations may contribute to transgenerational effects (Figure 5).

## 5. Discussion

Plant hormesis should be interpreted within the framework of physiological trade-offs between growth and defense. Because plants operate under resource constraints, exposure to low-intensity stress stimuli can trigger a redistribution of carbon, energy, and nutrients toward defense, repair, and acclimatization processes. Consequently, hormetic responses do not necessarily result in maximized biomass. While a moderate stimulus may promote growth under certain conditions, in other cases the predominant response may consist of strengthening defense systems, maintaining physiological homeostasis, or increasing the synthesis of secondary bioactive metabolites without a proportional increase in productivity. For this reason, biomass stimulation alone should not be considered a definitive criterion for identifying hormetic responses, as the adaptive outcome may manifest in other ways, such as greater stress tolerance, reproductive stability, or improved nutraceutical quality of plant tissues.

An alternative interpretation considers plant hormesis to be a particular form of acclimation that can be operationally identified as a biphasic growth response. This definition has the advantage of providing a clear and measurable criterion for detecting stimulation at low doses and inhibition at higher doses. However, growth responses alone may not fully reflect the biological consequences of stress exposure. An increase in biomass may coexist with reduced defensive or reproductive capacity, whereas beneficial responses may also manifest as greater stress tolerance, maintenance of reproduction, or other fitness-related traits without a proportional increase in growth. On the other hand, physiological acclimation may occur without a biphasic dose–response pattern and does not necessarily translate into greater survival or reproductive success; it may simply represent a physiological or metabolic adjustment that allows the organism to temporarily maintain its function under stress. Therefore, acclimation and hormesis should not be considered equivalent. For these reasons, we propose a broader interpretive framework in which the biphasic dose–response pattern may continue to be essential for identifying hormesis, while the biological significance of the response is evaluated according to the variable measured, its temporal and developmental context, the associated trade-offs, and its relationship to fitness.

Alternative explanations for low-dose stimulation should also be considered. Processes such as compensatory growth or physiological adjustment may, in some cases, form part of the mechanisms underlying a hormetic response when they are directly induced by a low-dose stressor and occur within a reproducible biphasic dose–response relationship. In contrast, responses that primarily reflect correction of nutrient deficiencies, developmental variation, or experimental variability should not automatically be interpreted as hormesis. Therefore, it is important to distinguish between nonspecific low-dose stimulation and compensatory or adaptive processes that are causally associated with the elicitor and contribute to a biologically meaningful outcome.

Hormesis should be considered a hierarchical and integrative biological process, rather than a simple two-phase dose–response pattern. This conceptual framework can be represented at three interconnected levels: (i) regulatory mechanisms, including signaling and transduction networks such as redox regulation, hormonal signaling, and gene expression control; (ii) measurable response variables, comprising physiological, biochemical, and molecular indicators such as enzyme activity, metabolite accumulation, and transcript abundance; and (iii) integrated fitness outcomes, representing the final biological consequence of the response, including survival, reproductive capacity, and offspring quality. Within this hierarchy, response variables do not reflect hormesis itself but, rather, serve as measurable indicators of the underlying regulatory processes that jointly determine the adaptive outcome. Thus, hormesis emerges as the net result of coordinated regulatory adjustments that, under appropriate environmental conditions, may translate into improved fitness-related outcomes, including enhanced survival, reproductive stability, or stress tolerance. From an evolutionary perspective, the cellular mechanisms activated in both plants and consumer organisms have evolved to promote fitness, understood as survival, reproduction, and the maintenance of physiological performance under changing environmental conditions. In this context, bioactive compounds produced by plants in response to stress can also play a role in xenohormesis-related processes by activating adaptive responses in the organisms that consume them.

Although xenohormesis provides a useful conceptual framework for interpreting interspecific signaling mediated by stress-induced metabolites, the available evidence remains limited. A major limitation of the current xenohormesis literature is that most studies demonstrate the bioactivity of isolated phytochemicals rather than a complete producer–consumer signaling sequence. Thus, the beneficial effects of plant metabolites in consumers should not be interpreted as direct evidence of xenohormesis unless stress-induced metabolite alteration in the producer, transfer to the consumer, and a subsequent adaptive outcome are experimentally demonstrated. This distinction is important, because general phytochemical bioactivity does not necessarily imply interspecific communication or anticipatory environmental signaling.

Despite advances in the study of hormesis, the regulatory mechanisms underlying it remain poorly understood. This lack of clarity makes it difficult to universally establish which variables are most appropriate for characterizing hormetic responses. Therefore, it is necessary to consider that response variables can vary depending on the type of stimulus that induces hormesis, as well as its (biogenic or natural) origin. Understanding these mechanisms enables the selection of more appropriate experimental variables for evaluation under controlled laboratory conditions, thereby contributing to a more precise characterization of the hormetic phenomenon and avoiding the measurement of uninformative indicators. Although methods have been proposed to estimate hormetic trade-offs in plants, current approaches remain limited in their ability to capture these trade-offs not only at the population level but also at the individual level, where resource allocation and adaptive responses are ultimately integrated. Under this framework, hormesis is viewed not as an individual response variable or merely as a biphasic dose–response pattern, but as an adaptive process emerging from the coordinated interaction of regulatory mechanisms whose ultimate significance lies in their contribution to fitness-related outcomes.

From an agricultural perspective, the practical relevance of hormesis lies in the fact that a low- or moderate-intensity stimulus can activate adaptive mechanisms that improve the plant’s ability to respond not only to the factor used as an elicitor, but also to other stress factors acting simultaneously in the environment. In this sense, the elicitor can be considered a tool to activate or strengthen the plant’s adaptive capacity, while the hormetic benefit is manifested in its subsequent performance under variable environmental conditions. Under this approach, fitness becomes particularly relevant to agriculture, as it allows two fundamental outcomes to be considered; namely, survival and reproductive capacity. The former is related to the ability of plants to maintain their functioning and survive under adverse conditions, such as drought, salinity, extreme temperatures, or diseases; the latter is reflected in reproductive performance, particularly in seed and fruit production. Therefore, a hormetic response of agronomic interest should not be evaluated based solely on an increase in a physiological variable or growth, but rather on its contribution to survival and yield under relevant environmental conditions. In addition, trade-offs among growth, defense, and secondary metabolism may favor the accumulation of bioactive metabolites without necessarily compromising yield, thereby contributing to both crop resilience and the functional quality of foods.

## 6. Future Research Directions

Future research should incorporate a dosimetric perspective, rather than limiting the experimental design to an approach based solely on increasing doses. For the evaluation of hormesis, the dose or intensity of the stress factor, the duration of exposure and, when appropriate, the frequency of exposure should be jointly considered. This perspective would allow for more precise characterization of the relationships between exposure conditions and organismal responses, ranging from adaptive responses to inhibitory or toxic effects. In addition, future studies should incorporate fitness as a final biological outcome of hormetic responses. To date, most experimental approaches have focused individually on physiological, biochemical, molecular, or growth-related variables, whereas the integration of these responses with fitness-related outcomes has received considerably less attention. Therefore, future experimental designs should relate response variables and associated trade-offs to survival and reproductive success, allowing for a more direct assessment of the adaptive significance of hormesis.

## 7. Conclusions

Hormesis is an adaptive process induced by a sublethal stimulus, in which the coordinated activation of regulatory mechanisms generates measurable physiological and biochemical changes. These responses do not constitute hormesis itself, but rather serve as indicators of the underlying process. Fitness-related outcomes provide the strongest evidence that a hormetic response has adaptive significance, taking into account the environmental context in which the response occurs. This framework complements the descriptive dose–response characterization of hormesis by providing an interpretive structure that links dose–response patterns with candidate regulatory mechanisms, measurable responses, and fitness-related outcomes.

A rigorous demonstration of the xenohormesis phenomenon still requires an explicit producer–consumer sequence, including an increasing elicitor dose, a stress-dependent alteration in the bioactive compound, and its influence on the fitness of the producer organism, as well as correlation with relevant variables in the consumer organism.

Although the available evidence remains limited, it may be cautiously inferred that organisms exposed to hormetic conditions and subsequently consumed by other organisms could potentially confer xenohormetic benefits. At present, much of the available evidence is based on the differential accumulation of bioactive compounds in organisms exposed to hormetic conditions and on the known biological effects of these compounds in consumers, rather than on a direct demonstration of the complete producer–consumer sequence.

## Figures and Tables

**Table 1:** Evolution of the major concepts and definitions of hormesis.

Author	Hormesis Definition	Main Emphasis	Contribution
Paracelsus (16th Century) [3]	“What is there that is not poison? All things are poison and nothing is without poison. Solely the dose determines that a thing is not a poison.”	Dose	The first evidence of the hormesis process established the importance of dose in determining whether exposure produces a beneficial or harmful effect.
Arndt–Schulz’s law [4,5]	“For every substance, small doses stimulate, moderate doses inhibit, large doses kill.”	Dose–response	Describes the overall response across the entire dose range and relates it to the biological response, including stimulation at low doses.
Southam and Ehrlich [6]	“A stimulatory effect of subinhibitory concentrations of any toxic substance on any organism.”	Low-dose stimulation	Explicit early formulation of hormesis as stimulation caused by subinhibitory concentrations of a toxic substance.
Stebbing [7]	Hormesis is the name given to the stimulatory effects caused by low levels of potentially toxic agents.	Low-level stimulation	Emphasizes stimulation produced by low levels of toxic agents.
Christiani and Zhou [8]	Hormesis is a dose–response phenomenon characterized by either a U-shaped or an inverted U-shaped dose response depending on the different endpoints measured.	Biphasic dose–response	Hormesis is defined in terms of the shape of the dose–response relationship and recognizes that this pattern may vary depending on the endpoint being evaluated.
Calabrese and Baldwin [9]	Hormesis should be considered an adaptive response characterized by biphasic dose responses of generally similar quantitative features with respect to amplitude and range of the stimulatory response that are either directly induced or the result of compensatory biological processes following an initial disruption in homeostasis.	Adaptation and compensation	Considers hormesis beyond a descriptive dose–response relationship and incorporates adaptive and compensatory biological processes.
Mattson [10]	“A process in which exposure to a low dose of a chemical agent or environmental factor that is damaging at higher doses induces an adaptive beneficial effect on the cell or organism”.	Adaptive benefit	The adaptive response is once again incorporated into the definition.
Mattson and Calabrese [11]	Hormesis describes any process in which a cell, organism, or group of organisms exhibits a biphasic response to exposure to increasing amounts of a substance or condition (e.g., chemical, sensory stimulus, or metabolic stress); typically, low-dose exposures elicit a stimulatory or beneficial response, whereas high doses cause inhibition or toxicity.	Biphasic response across biological levels	Broadens the concept to different biological levels and types of stressors while retaining the biphasic dose–response pattern as a central characteristic.
Vargas-Hernández [2]	“Hormesis is a nonlinear adaptive process through which cells, and consequently living organisms, respond to an increasing range of doses and time of exposure to a stressor. At low or moderate levels and/or short exposure times, the stimulus activates signaling and transduction networks that modulate gene expression, redox metabolism, and the synthesis of protective molecules, promoting biochemical and physiological adjustments and reaching a maximum value of the variable of interest associated with adaptation. Once a critical threshold of dose or exposure time is exceeded, defense mechanisms become saturated or dysregulated, and the beneficial response declines, leading to toxic or inhibitory effects”	Nonlinear adaptation, mechanisms, dose and exposure time	Extends the adaptive concept by explicitly incorporating dose and exposure time, and molecular mechanisms from adaptive responses to toxicity.

**Table 2:** Effects of various stress factors on physiological responses and bioactive compound production in plants.

Organism	Stressor/Dose	Developmental Stage	Exposition Time/ Experiment Time	Physiological Effect	Bioactive Compound Production	Reference
*Zea mays*	Cd and Pb (0, 0.5, 1, 5, 10, 50, 100 μM)	7-day-old seedlings	24 h/14 days	Increased growth; increased IAA concentration.	Increased chlorophyll and flavonol contents.	[21]
*Lonicera japonica*	Cd (0, 2.5, 5, 10, 25, 50, 100 mg/kg)	Rooted seedlings	90 days	Increased plant biomass; stomatal conductance, transpiration rate, net photosynthetic rate; PSII potential efficiency, effective quantum yield, and photochemical quenching coefficient.	Increased chlorophyll (a, b, a + b) and carotenoids.	[23]
*Lonicera japonica*	Cd (0, 5, 25 mg/L); additional experimental factor: electric field (0, 1, 2, 3 V/cm)	8-week-old plants	8 weeks	Increased root and leaf biomass dry weight.	Increased chlorophyll a, b, total chlorophyll, and carotenoids in leaves.	[22]
*Mentha × piperita*	Cd (0, 0.8, 1.6, 3, 6.5 mg/L)	Young plants	7 days	Increased fresh and dry weight of leaves, shoots, and roots.	Increased total flavonoid and total phenolic contents.	[25]
*Poa annua* L.	Cr (0, 5, 10, 25, 50, 100 μM) and Cd (0, 0.5, 0.75, 1, 1.5, 2 µM)	Seedlings	4 weeks	Increased root, shoot dry weight, number of nodes, and leaf area.	Increased total photosynthetic pigments (chlorophyll a, b, total carotenoids).	[26]
*Pisum sativum*	Pb(NO_3_)_2_ (0.075, 0.5 mM) with and without aphids	5-day-old seedlings	11 days/6 h	Accumulation of abscisic acid (ABA) and salicylic acid (SA).	Flavonoid biosynthesis, including the phytoalexin pisatin.	[27]
*Corchorus olitorius*	Pb (30, 60, 120 mg/kg soil)	15-day-old seedlings	45 days/30 days	Increased fresh weight, dry weight, plant height, leaf area, and number of leaves.	Increased chlorophyll a, b, total phenolic compounds, and total anthocyanins.	[28]
*Pisum sativum*	Pb(NO_3_)_2_ (0, 0.075, 0.5 mM) with and without aphids	5-day-old seedlings	66 h/0, 24, 48, and 72 h	High expression of *JAR1*, *OPR1*, and *ACS3*; accumulation of ABA and SA.	Accumulation of defensive metabolites.	[29]
*Triticum* spp.	Zn (0, 10, 25, 50, 100, 300, 500, 800, 1000 μM)	Two-leaf-stage seedlings	14 days	Increased root, shoot growth, photosynthetic rate, stomatal conductance, intercellular CO_2_ concentration, and transpiration rate.	Increased chlorophyll a, chlorophyll b, carotenoids, and total chlorophyll.	[30]
*Brassica oleracea*	UV-B (0, 5, 10, 15 kJ/m^2^)	During germination 3, 5, 7, 10 days	10 days	Increased antioxidant activity.	Increased phenolic content (including hydroxycinnamic acids); increased kaempferol-3,7-di-O-glucoside; increased gallic acid hexoside I and gallic acid; increased glucoraphanin and 4-hydroxy-glucobrassicin.	[31]
*Fragaria × ananassa*	UV-C (9.6, 15, 29.4 kJ/m^2^)	Potted plants	7 weeks	Expression of flavonoid pathway structural genes (*FaCHS1*, *FaCHI*, *FaFHT*, *FaDFR*, *FaFLS*, *FaFGT*).	Accumulation of phenolics (cyanidin-3-glucoside; pelargonidin-3-glucoside/rutinoside; quercetin and kaempferol glucosides and glucuronides; ellagic acid).	[32]
*Brassica oleracea*	UV-C (0, 1.2, 3.0 kJ/m^2^)	Florets	27 days	Delayed yellowing and reduced weight loss; overexpression of phenylalanine N-hydroxylase, tryptophan N-hydroxylase, dihomo-methionine N-hydroxylase, flavonoid monooxygenase, chalcone synthase, coumarate ligase.	Increased total glucobrassicins and 4-hydroxyglucobrassicin; higher hydroxycinnamic acids.	[33]
*Vitis vinifera* L.	UV-C (0, 2, 3 kJ/m^2^)	Harvested plant material	17.5 or 26.3 min	Increased spectrophotometric color.	Increased total anthocyanin monomers, pyranoanthocyanins, direct condensation products, and acetaldehyde-mediated condensation products.	[34]
*Capsicum annuum* L.	Silicon (0, 60, 125, 250 mg/L)	37-day-old seedlings	28 days	Improved leaf area, increased fresh and dry biomass, leaf and stem weight, and total soluble sugars.	Increased chlorophyll a, b and amino acid concentration.	[35]
*Brassica campestris* L.	Tetracycline (0–50 mg/L)	Late seedling to vegetative stage (3–4 weeks) after sowing)	6 weeks	Increased fresh weight and leaf number.	No differences in chlorophylls or carotenoids against control.	[36]
*Dracocephalum kotschyi*	NiO NPs (0, 50, 100, 1000, 2500 mg/L)	Seedlings	3 weeks	Increased shoot height and weight (~33%); increased SOD, POD, CAT, and APX.	Stimulated chlorophyll, carotenoid, and anthocyanin content.	[24]

**Table 3:** Plant bioactives produced during hormesis: roles in plants and xenohormetic potential.

Classification/ Compound	Plants (Hormesis)					Humans (Xenohormesis)	
	Species	Stressor	Adaptation Response	Beneficial Dose/Concentration	Reference	Therapeutic Potential/Potential Relevance to Xenohormesis	Type of Evidence
**Terpenoids** Carotenoids	*Dracocephalum moldavica*	0, 50, 100, and 200 mg/L of TiO_2_ NPs and 0, 50, and 100 mM NaCl	Lowered H_2_O_2_ content and increased antioxidant enzyme activities	100 mg/L–0.81 mg/g	[37]	Potential protective effects against cancer [38], cardiovascular diseases [39], macular degeneration [40], and age-related degenerative conditions [41].	Proposed therapeutic potential [38]; observational/human health evidence [39]; cell-based, animal, and human/clinical evidence [40]; preclinical and human evidence/proposed therapeutic potential [41].
	*Salvinia natans* L.	Cu2+ + glyphosate mixtures at 0 + 0, 0.2 + 1, 1 + 5, 5 + 25, 8 + 50, 10 + 75 mg/L	Increased H_2_O_2_ content and increased antioxidant enzyme activities	≤5 mg/L of glyphosate	[42]		
**Flavonoids** Total flavonoids, anthocyanins, flavonols, kaempferol-3,7-di-o-glucoside, quercetin glucoside, pelargonidin-3-glucoside, pelargonidin-3-rutinoside, cyanidin-3-glucoside, pisatin	*Mentha piperita*	0, 0.8, 1.6, 3 and 6.5 mg/L of Cd	Enhanced antioxidant activity by avoiding oxidative damages caused by Cd ion, while high level of Cd stress just induced a temporary increase in antioxidant activity	1.6 mg/L	[25]	Antioxidant and anti-inflammatory activities, potential neuroprotective, cardioprotective, antidiabetic, anticancer, antitumor, metabolic effects, and neurological disorders [43,44,45].	Cell-based/preclinical evidence and proposed therapeutic potential [44]; cell-based/mechanistic and animal/preclinical evidence [45]; primary animal study (in vivo) [46].
	*Bupleurum chinense* DC	0, 50, 100, 125, 150, and 200 Gy using carbon irradiation	Enhanced production of secondary metabolites	50 Gy of CIB	[43]		
**Phenolic compounds** Total phenolics, hydroxycinnamic acids, gallic acid, gallic acid hexoside, ellagic acid	*Thymus vulgaris*	0, 1, 3, and 5 Gy using gamma irradiation	ROS generation and antioxidant enzyme activities revealed the complicated and multifaceted biochemical response	1 Gy of gamma irradiation	[47]	Antioxidant and anti-inflammatory activities, potential anticancer/antitumor, antimicrobial, cardioprotective, anti-atherogenic, cardiometabolic modulation, and neuroprotective effects [48,49].	Preclinical evidence and proposed therapeutic potential [48]; cell-based, animal, and human evidence [49].
	*Ocimum basilicum*	0, 5, 10, 20, 30, 40, 60, 80, 100, 120, and 200 mg/L of TiO_2_	Stress exposure simultaneously modulates antioxidant mechanisms and specialized metabolism	20–30 mg/L-60%	[50]		
**Glucosinolates** Total glucobrassicins, glucoraphanin, 4-hydroxy-glucobrassicin	*Brassica napus* L.	25, 50, 75, and 100 mg/L of plant-based Zn-Se NCs	Enhanced plant growth and antioxidant levels	25 mg/L	[51]	Potential anticancer, antioxidant and anti-inflammatory, cardiovascular, neuroprotective, modulation of biochemical parameters, improvement of blood glucose and lipid profiles [52,53].	Cell-based, animal, and human evidence [52]; preclinical and human evidence (systematic review)/proposed therapeutic potential [53].
	*Brassica juncea*	UV-C light supplementation (0.3 kJ m^−2^, 254 nm): one pulse and three pulses	Improved growth parameters and decreased phenolic content and antioxidant activity	One pulse	[54]		

**Table 4:** Candidate regulatory mechanisms, measurable response variables, and potential fitness-related outcomes associated with plant hormesis.

Plant Stress	Candidate Regulatory Mechanisms	Response Variables	Hormesis Response	Stressor	Plants Species
Salt Stress	Ion homeostasis, osmotic stress pathways, hormone signaling, cytoskeleton and cell wall composition [63,64,65].	Cytosolic and vacuolar ion concentrations (Na^+^, K^+^, Ca^2+^, Cl^−^), Na^+^/K^+^ ratio, activity or expression of ion transporters and channels (e.g., HKT, SOS1, NHX, CAX), membrane potential, ion fluxes, electrolyte leakage [66,67,68].	It maintains survival and reproductive stability under salt stress [69].	0, 50, and 100 mM NaCl; 0, 50, 100, 150, 200, 250 mM NaCl	*Moringa oleifera* [70]
Temperature Stress	Metabolic rearrangements, regulatory network activation, gene expression changes [71].	Soluble sugars/starch, amino acids (e.g., proline, GABA), organic acids (malate, citrate). C:N ratio, regulation and signaling, phytohormones (ABA, auxins, JA, SA, ethylene), signal metabolites (trehalose-6-phosphate, GABA), kinase activity (SnRK1, TOR, MAPKs), redox state (NAD(P)H, glutathione), Ca^2+^ dynamics, stress response genes (LEA, HSPs), transcription factors (bZIP, MYB, NAC, WRKY), epigenetic markers (DNA methylation, histones) [72,73,74,75,76,77,78,79].	Reduce mortality and reproductive failures, enhance fitness or related variables [80,81,82].	12.5–27.5 C; 18–30 C	*Phaseolus vulgaris* L.; *Quercus myrsinaefolia*: *Populus tremula 9 P. tremuloides*; *Betula hybrid* “Royal Frost” [81]
UV Stress	UVR8 signaling pathway, antioxidant and photoprotective systems, stimulation of phenylpropanoid pathway, DNA repair (photolyases, NER), chloroplast retrograde signaling [83,84].	Flavonoids and other phenolics Anthocyanins ROS levels (H_2_O_2_, O_2_•^−^) Antioxidant enzyme activity (SOD, CAT, APX, POD), PSII efficiency (Fv/Fm), DNA damage markers (CPDs), expression of UV-responsive genes [85,86,87,88].	Increased tolerance to subsequent stress and reproductive success under moderate UV exposure, activation of defense mechanism [87,89].	UV-C (0, 2, 3 kJ/m^2^); UV-C (0, 1.2, 3.0 kJ/m^2^); UV-C (9.6, 15, 29.4 kJ/m^2^); UV-B (0, 5, 10, 15 kJ/m^2^).	*Vitis vinifera* L. [34]; *Brassica oleracea* [33]; *Fragaria × ananassa* [32].
Disease/Infection Stress	Activation of innate immunity (PTI/ETI), SA, JA, and ethylene signaling networks, systemic acquired resistance (SAR), induced systemic resistance (ISR), ROS and Ca^2+^ signaling, cell wall reinforcement and callose deposition [90,91,92,93,94].	PR1, PR2, PR5, phytoalexins, SA, JA, ethylene levels, ROS burst, callose and lignin deposition, expression of defense-related genes, microbiome/pathobiome shifts [91,95,96].	Enhanced resistance to future infections, reduced disease severity, maintenance of reproductive output despite pathogen pressure [14].	Xanthomonas campestris; Ralstonia solanacearum; *Botrytis cinerea*, *Fusarium virguliforme*, *Globisporangium ultimum*, *Globisporangium. irregulare*, *Lasiodiplodia theobromae*, *Magnaporthe oryzae*, *Sclerotinia sclerotiorum*, *Sclerotinia homoeocarpa*, and *Pythium aphanidermatum* [97].	*Capsicum annuum*; *Solanum lycopersicum* [98].

**Table 5:** Candidate regulatory mechanisms, measurable response variables, and potential fitness-related outcomes associated with xenohormesis.

Xenohormetic Signal	Candidate Regulatory Mechanisms	Response Variables	Xenohormetic Response
Flavonoids (quercetin, kaempferol, catechins)	Antioxidant signaling pathways (Nrf2), AMPK–SIRT1 metabolic pathways, NF-κB regulation, mitochondrial stress responses [99,100].	SOD, CAT, GPx activity, ROS/redox balance, stress response gene expression, mitochondrial biogenesis, ATP/AMP ratio [101,102,103,104].	Increased cellular stress resistance, improved metabolic efficiency, enhanced survival under environmental stress [17].
Phenolic compounds (phenolic acids, tannins, lignans)	Nrf2, ARE signaling, detoxification pathways, inflammatory modulation, regulation of cellular stress response [100,105].	GST, CYP450 activity, redox biomarkers (glutathione levels, NAD(P)H), antioxidant genes and oxidative damage markers [106,107,108].	Improved oxidative stress tolerance and maintenance of physiological homeostasis [109,110].
Glucosinolates and isothiocyanates	Cellular defense and detoxification, induction of phase II detoxification enzymes, immune signaling pathways [111,112].	GST, NQO1 expression, antioxidant, immune signaling molecules, cellular stress markers [113,114].	Enhanced detoxification capacity and resilience to chemical and environmental stress [111].
Terpenoids (carotenoids, monoterpenes, sesquiterpenes)	Oxidative stress signaling, hormonal and metabolic pathways, antioxidant defense systems [115].	ROS scavenging, lipid peroxidation markers, antioxidant enzyme activity, stress-responsive gene expression [116,117].	Enhanced tolerance to environmental stress, antiaging [118,119].

## Data Availability

No new data were created or analyzed in this study. Data sharing is not applicable to this article.

## Abbreviations

The following abbreviations are used in this manuscript:

ABA	Abscisic acid
ACC	1-aminocyclopropane-1-carboxylic acid
*ACS3*	1-aminocyclopropane-1-carboxylate synthase 3
APX	Ascorbate peroxidase
CAT	Catalase
Cd	Cadmium
CO_2_	Carbon dioxide
Cr	Chromium
CYP450	Cytochrome P450 enzymes
*FaCHI*	*Fragaria × ananassa* chalcone isomerase
*FaCHS1*	*Fragaria × ananassa* chalcone synthase 1
*FaDFR*	*Fragaria × ananassa* dihydroflavonol 4-reductase
*FaFGT*	*Fragaria × ananassa* flavonoid glycosyltransferase
*FaFHT*	*Fragaria × ananassa* flavonoid hydroxylase (usage may vary by author)
*FaFLS*	*Fragaria × ananassa* flavonol synthase
IAA	Indole-3-acetic acid
JA	Jasmonic acid
*JAR1*	Jasmonic acid–amido synthetase 1
MeJA	Methyl jasmonate
NiO NPs	Nickel oxide nanoparticles
NPs	Nanoparticles
NaCl	Sodium chloride
*OPR1*	12-oxophytodienoate reductase 1
Pb	Lead
Pb(NO_3_)_2_	Lead nitrate
POD	Peroxidase
PSII	Photosystem II
qP	Photochemical quenching coefficient
ROS	Reactive oxygen species
SA	Salicylic acid
SOD	Superoxide dismutase
UV-B	Ultraviolet-B radiation
UV-C	Ultraviolet-C radiation
ΦPSII	Effective quantum yield of Photosystem II
Zn	Zinc
